# 5-[(2-Chloro-4-nitro­anilino)methyl­idene]-2,2-dimethyl-1,3-dioxane-4,6-dione

**DOI:** 10.1107/S160053681100095X

**Published:** 2011-01-15

**Authors:** Xian-Qiu Lan, Xiao-Feng Zhang, Ying-Hong Yang, You-Fu Luo

**Affiliations:** a Department of Pharmaceutical and Bioengineering, School of Chemical Engineering, Sichuan University, Chengdu 610065, People’s Republic of China; b State Key Laboratory of Biotherapy, West China Hospital, Sichuan University, Chengdu 610041, People’s Republic of China

## Abstract

In the title compound, C_13_H_11_ClN_2_O_6_, the dihedral angles between the benzene ring and the amino­methyl­ene unit and between the amino­methyl­ene group and the dioxane ring are 8.19 (14) and 1.39 (17)°, respectively. The dioxane ring has a half-boat conformation, in which the C atom between the dioxane O atoms is 0.662 (4)Å out of the plane through the remaining ring atoms. Intra­molecular N—H⋯O and N—H⋯Cl inter­actions occur.

## Related literature

For the synthesis of related compounds, see: Cassis *et al.* (1985 ▶). For the biological activity of related compounds, see: Griera *et al.* (1997 ▶); Darque *et al.* (2009 ▶).
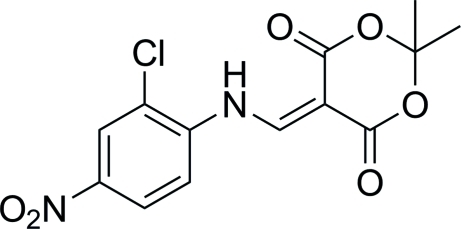


## Experimental

### 

#### Crystal data

C_13_H_11_ClN_2_O_6_
*M* *_r_* = 326.69
Monoclinic, 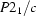

*a* = 13.5850 (5) Å
*b* = 5.04379 (14) Å
*c* = 21.0272 (7) Å
β = 104.427 (4)°
*V* = 1395.35 (8) Å^3^
*Z* = 4
Mo *K*α radiation
μ = 0.31 mm^−1^
*T* = 293 K
0.40 × 0.40 × 0.30 mm

#### Data collection

Oxford Diffraction Xcalibur Eos diffractometer
Absorption correction: multi-scan (*CrysAlis PRO*; Oxford Diffraction, 2010) ▶ *T*_min_ = 0.984, *T*_max_ = 1.0
6136 measured reflections
2853 independent reflections
2141 reflections with *I* > 2σ(*I*)
*R*_int_ = 0.021

#### Refinement

*R*[*F* ^2^ > 2σ(*F* ^2^)] = 0.041
*wR*(*F* ^2^) = 0.104
*S* = 1.04
2853 reflections
201 parameters
H-atom parameters constrained
Δρ_max_ = 0.17 e Å^−3^
Δρ_min_ = −0.29 e Å^−3^

### 

Data collection: *CrysAlis PRO* (Oxford Diffraction, 2010) ▶; cell refinement: *CrysAlis PRO* ▶; data reduction: *CrysAlis PRO* ▶; program(s) used to solve structure: *SHELXS97* (Sheldrick, 2008 ▶); program(s) used to refine structure: *SHELXL97* (Sheldrick, 2008 ▶); molecular graphics: *OLEX2* (Dolomanov *et al.*, 2009 ▶); software used to prepare material for publication: *OLEX2*.

## Supplementary Material

Crystal structure: contains datablocks I, global. DOI: 10.1107/S160053681100095X/bq2269sup1.cif

Structure factors: contains datablocks I. DOI: 10.1107/S160053681100095X/bq2269Isup2.hkl

Additional supplementary materials:  crystallographic information; 3D view; checkCIF report

## Figures and Tables

**Table 1:** Hydrogen-bond geometry (Å, °)

*D*—H⋯*A*	*D*—H	H⋯*A*	*D*⋯*A*	*D*—H⋯*A*
N2—H2⋯Cl1	0.86	2.46	2.9328 (15)	115
N2—H2⋯O3	0.86	1.99	2.670 (2)	136

**Fig. 1.**
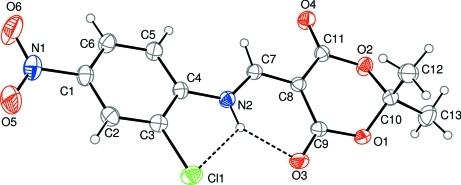
The molecular structure of the title compound, with displacement ellipsoids drawn at the 30% probability level. The intramolecular hydrogen bonds are shown as a dashed lines.

## supplementary crystallographic information

### Comment

The 4(1*H*)quinolone are of great importance owing to their wide biological properties (Griera *et al.*, 1997; Darque *et al.*,2009). 5-{[(2-Chloro-4-nitrophenyl)amino]methylene}-2,2-dimethyl-1,3-dioxane- 4,6-dione is one of the key intermediates in our synthetic investigations of new 4(1*H*)quinolone derivatives. We report here its crystal structure. The title compound is approximately planar, the dihedral angles between the benzene ring and the aminomethylene unit and between the aminomethylene group and the dioxane ring are 8.19 (14)° and 1.39 (17)°, respectively. The dioxane ring has a half-boat conformation, in which the C atom between the dioxane O atoms is 0.6615 (35) Å out of the plane (Figure 1.). In the molecule, there are intramolecular N—H···O and N—H···Cl interactions (Table 1.).

### Experimental

An ethanol solution (50 ml) of 2,2–dimethyl–1,3–dioxane–4,6–dione (1.44 g, 10 mmol) and triethoxymethane (1.78 g, 12 mmol) was heated to reflux for 2.5 h, then the 2-chloro-4-nitroaniline(1.72 g, 10 mmol) was added into the solution. The mixture was heated under reflux for another 8 h and then filtered. The precipitate was recrystallized from ethanol, giving the title compound. Crystals suitable for X-ray analysis were obtained by slow evaporation from a solution of ethanol.

### Refinement

The H-atom of N was located in a difference Fourier map and free refined: N—H = 0.86 Å. The other H atoms were positioned geometrically and refined using a riding model, with C—H = 0.93 Å and U*_iso_*(H) = 1.2U*_eq_*(C) for aromatic,*C*—H = 0.96 Å and U*_iso_*(H) = 1.5U*_eq_*(C) for methyl H atoms.

### Crystal data

**Table:**

C_13_H_11_ClN_2_O_6_	*F*(000) = 672
*M**_r_* = 326.69	*D*_x_ = 1.555 Mg m^−^^3^
Monoclinic, *P*2_1_/*c*	Mo *K*α radiation, λ = 0.7107 Å
Hall symbol: -P 2ybc	Cell parameters from 2432 reflections
*a* = 13.5850 (5) Å	θ = 3.0–29.2°
*b* = 5.04379 (14) Å	µ = 0.31 mm^−^^1^
*c* = 21.0272 (7) Å	*T* = 293 K
β = 104.427 (4)°	Block, colorless
*V* = 1395.35 (8) Å^3^	0.40 × 0.40 × 0.30 mm
*Z* = 4	

### Data collection

**Table:**

Oxford Diffraction Xcalibur Eos diffractometer	2853 independent reflections
Radiation source: fine-focus sealed tube	2141 reflections with *I* > 2σ(*I*)
graphite	*R*_int_ = 0.021
Detector resolution: 16.0874 pixels mm^-1^	θ_max_ = 26.4°, θ_min_ = 3.1°
ω scans	*h* = −16→16
Absorption correction: multi-scan (*CrysAlis PRO*; Oxford Diffraction, 2010)	*k* = −6→6
*T*_min_ = 0.984, *T*_max_ = 1.0	*l* = −25→26
6136 measured reflections	

### Refinement

**Table:**

Refinement on *F*^2^	Primary atom site location: structure-invariant direct methods
Least-squares matrix: full	Secondary atom site location: difference Fourier map
*R*[*F*^2^ > 2σ(*F*^2^)] = 0.041	Hydrogen site location: inferred from neighbouring sites
*wR*(*F*^2^) = 0.104	H-atom parameters constrained
*S* = 1.04	*w* = 1/[σ^2^(*F*_o_^2^) + (0.0478*P*)^2^ + 0.1291*P*] where *P* = (*F*_o_^2^ + 2*F*_c_^2^)/3
2853 reflections	(Δ/σ)_max_ < 0.001
201 parameters	Δρ_max_ = 0.17 e Å^−^^3^
0 restraints	Δρ_min_ = −0.29 e Å^−^^3^

### Special details

**Table:**

Experimental. CrysAlisPro, Oxford Diffraction Ltd., Version 1.171.34.40 (release 27-08-2010 CrysAlis171 .NET) Empirical absorption correction using spherical harmonics, implemented in SCALE3 ABSPACK scaling algorithm.
Geometry. All e.s.d.'s (except the e.s.d. in the dihedral angle between two l.s. planes) are estimated using the full covariance matrix. The cell e.s.d.'s are taken into account individually in the estimation of e.s.d.'s in distances, angles and torsion angles; correlations between e.s.d.'s in cell parameters are only used when they are defined by crystal symmetry. An approximate (isotropic) treatment of cell e.s.d.'s is used for estimating e.s.d.'s involving l.s. planes.
Refinement. Refinement of *F*^2^ against ALL reflections. The weighted *R*-factor *wR* and goodness of fit *S* are based on *F*^2^, conventional *R*-factors *R* are based on *F*, with *F* set to zero for negative *F*^2^. The threshold expression of *F*^2^ > σ(*F*^2^) is used only for calculating *R*-factors(gt) *etc*. and is not relevant to the choice of reflections for refinement. *R*-factors based on *F*^2^ are statistically about twice as large as those based on *F*, and *R*- factors based on ALL data will be even larger.

### Fractional atomic coordinates and isotropic or equivalent isotropic displacement parameters (Å^2^)

**Table:**

	*x*	*y*	*z*	*U*_iso_*/*U*_eq_	
Cl1	0.11303 (4)	0.78022 (10)	0.26632 (2)	0.05062 (18)	
O1	−0.23464 (10)	0.7514 (2)	0.04172 (7)	0.0446 (4)	
O2	−0.24014 (10)	0.4073 (3)	−0.03503 (6)	0.0469 (4)	
O3	−0.09905 (11)	0.8273 (3)	0.12205 (7)	0.0517 (4)	
O4	−0.10943 (10)	0.1444 (3)	−0.03156 (7)	0.0509 (4)	
O5	0.46457 (14)	0.3369 (4)	0.35706 (9)	0.0855 (6)	
O6	0.48067 (14)	0.0203 (4)	0.29216 (9)	0.0910 (6)	
N1	0.43369 (14)	0.2060 (4)	0.30716 (10)	0.0592 (5)	
N2	0.05320 (11)	0.4776 (3)	0.14363 (7)	0.0360 (4)	
H2	0.0263	0.6155	0.1565	0.043*	
C1	0.33473 (15)	0.2739 (4)	0.26352 (9)	0.0432 (5)	
C2	0.27826 (15)	0.4698 (4)	0.28308 (9)	0.0431 (5)	
H2A	0.3024	0.5569	0.3229	0.052*	
C3	0.18530 (14)	0.5341 (3)	0.24251 (9)	0.0377 (4)	
C4	0.14835 (13)	0.4042 (3)	0.18250 (8)	0.0337 (4)	
C5	0.20803 (15)	0.2071 (4)	0.16447 (9)	0.0421 (5)	
H5	0.1847	0.1193	0.1247	0.051*	
C6	0.30095 (15)	0.1410 (4)	0.20485 (10)	0.0455 (5)	
H6	0.3404	0.0086	0.1928	0.055*	
C7	−0.00122 (13)	0.3622 (3)	0.08932 (8)	0.0336 (4)	
H7	0.0254	0.2132	0.0736	0.040*	
C8	−0.09479 (13)	0.4525 (3)	0.05552 (8)	0.0328 (4)	
C9	−0.14016 (14)	0.6847 (3)	0.07640 (9)	0.0373 (4)	
C10	−0.29653 (14)	0.5492 (4)	0.00333 (10)	0.0437 (5)	
C11	−0.14546 (14)	0.3172 (4)	−0.00500 (9)	0.0359 (4)	
C12	−0.33222 (18)	0.3613 (4)	0.04848 (13)	0.0656 (7)	
H12B	−0.3779	0.2337	0.0229	0.098*	
H12C	−0.3668	0.4590	0.0756	0.098*	
H12A	−0.2748	0.2713	0.0758	0.098*	
C13	−0.38047 (18)	0.6932 (5)	−0.04496 (12)	0.0695 (7)	
H13B	−0.3514	0.8249	−0.0678	0.104*	
H13C	−0.4245	0.7774	−0.0218	0.104*	
H13A	−0.4188	0.5687	−0.0760	0.104*	

### Atomic displacement parameters (Å^2^)

**Table:**

	*U*^11^	*U*^22^	*U*^33^	*U*^12^	*U*^13^	*U*^23^
Cl1	0.0589 (4)	0.0482 (3)	0.0464 (3)	0.0072 (2)	0.0162 (3)	−0.0079 (2)
O1	0.0400 (8)	0.0317 (6)	0.0572 (9)	0.0054 (6)	0.0025 (7)	−0.0053 (6)
O2	0.0393 (8)	0.0582 (8)	0.0405 (8)	0.0068 (6)	0.0048 (6)	−0.0090 (7)
O3	0.0527 (9)	0.0407 (7)	0.0556 (9)	0.0058 (6)	0.0018 (7)	−0.0167 (7)
O4	0.0477 (9)	0.0585 (9)	0.0464 (8)	0.0064 (7)	0.0118 (7)	−0.0192 (7)
O5	0.0679 (12)	0.1027 (14)	0.0660 (11)	0.0073 (10)	−0.0210 (10)	−0.0129 (11)
O6	0.0689 (12)	0.1144 (15)	0.0783 (13)	0.0444 (12)	−0.0031 (10)	0.0001 (11)
N1	0.0447 (11)	0.0748 (13)	0.0522 (12)	0.0057 (10)	0.0013 (9)	0.0098 (10)
N2	0.0349 (8)	0.0370 (8)	0.0364 (9)	0.0019 (7)	0.0092 (7)	−0.0035 (7)
C1	0.0369 (11)	0.0526 (12)	0.0382 (11)	0.0015 (9)	0.0060 (9)	0.0074 (9)
C2	0.0462 (12)	0.0481 (11)	0.0327 (10)	−0.0039 (9)	0.0051 (9)	−0.0018 (9)
C3	0.0409 (11)	0.0385 (10)	0.0358 (10)	−0.0014 (8)	0.0136 (9)	0.0005 (8)
C4	0.0317 (10)	0.0373 (9)	0.0333 (10)	−0.0022 (7)	0.0107 (8)	0.0030 (8)
C5	0.0404 (11)	0.0500 (11)	0.0357 (11)	0.0028 (9)	0.0090 (9)	−0.0051 (9)
C6	0.0412 (11)	0.0497 (11)	0.0467 (12)	0.0067 (9)	0.0128 (9)	−0.0008 (10)
C7	0.0340 (10)	0.0351 (9)	0.0340 (10)	−0.0001 (8)	0.0130 (8)	−0.0005 (8)
C8	0.0342 (10)	0.0314 (9)	0.0344 (10)	−0.0008 (7)	0.0115 (8)	−0.0017 (8)
C9	0.0388 (10)	0.0308 (9)	0.0416 (11)	−0.0010 (8)	0.0086 (9)	0.0004 (8)
C10	0.0365 (10)	0.0413 (10)	0.0529 (12)	0.0015 (8)	0.0099 (9)	−0.0124 (9)
C11	0.0339 (10)	0.0400 (10)	0.0356 (10)	−0.0012 (8)	0.0120 (8)	−0.0014 (9)
C12	0.0596 (15)	0.0528 (13)	0.0971 (19)	−0.0072 (11)	0.0431 (14)	−0.0094 (13)
C13	0.0470 (14)	0.0787 (17)	0.0703 (16)	0.0174 (12)	−0.0089 (12)	−0.0148 (13)

### Geometric parameters (Å, °)

**Table:**

Cl1—C3	1.7321 (19)	C3—C4	1.399 (2)
O1—C9	1.351 (2)	C4—C5	1.394 (2)
O1—C10	1.436 (2)	C5—H5	0.9300
O2—C10	1.434 (2)	C5—C6	1.375 (3)
O2—C11	1.362 (2)	C6—H6	0.9300
O3—C9	1.218 (2)	C7—H7	0.9300
O4—C11	1.203 (2)	C7—C8	1.371 (2)
O5—N1	1.222 (2)	C8—C9	1.442 (2)
O6—N1	1.218 (2)	C8—C11	1.457 (2)
N1—C1	1.467 (3)	C10—C12	1.505 (3)
N2—H2	0.8600	C10—C13	1.511 (3)
N2—C4	1.396 (2)	C12—H12B	0.9600
N2—C7	1.330 (2)	C12—H12C	0.9600
C1—C2	1.375 (3)	C12—H12A	0.9600
C1—C6	1.378 (3)	C13—H13B	0.9600
C2—H2A	0.9300	C13—H13C	0.9600
C2—C3	1.375 (2)	C13—H13A	0.9600
			
O1—C9—C8	117.28 (15)	C5—C4—N2	123.12 (16)
O1—C10—C12	109.17 (17)	C5—C4—C3	118.49 (16)
O1—C10—C13	105.94 (16)	C5—C6—C1	118.96 (19)
O2—C10—O1	110.53 (15)	C5—C6—H6	120.5
O2—C10—C12	109.99 (15)	C6—C1—N1	119.67 (19)
O2—C10—C13	106.34 (17)	C6—C5—C4	120.78 (17)
O2—C11—C8	115.76 (16)	C6—C5—H5	119.6
O3—C9—O1	117.76 (16)	C7—N2—H2	115.8
O3—C9—C8	124.94 (17)	C7—N2—C4	128.46 (15)
O4—C11—O2	118.24 (17)	C7—C8—C9	121.58 (16)
O4—C11—C8	125.91 (17)	C7—C8—C11	118.19 (16)
O5—N1—C1	118.4 (2)	C8—C7—H7	118.5
O6—N1—O5	123.2 (2)	C9—O1—C10	118.10 (13)
O6—N1—C1	118.38 (19)	C9—C8—C11	120.12 (16)
N2—C4—C3	118.39 (16)	C10—C12—H12B	109.5
N2—C7—H7	118.5	C10—C12—H12C	109.5
N2—C7—C8	123.03 (16)	C10—C12—H12A	109.5
C1—C2—H2A	120.7	C10—C13—H13B	109.5
C1—C2—C3	118.59 (17)	C10—C13—H13C	109.5
C1—C6—H6	120.5	C10—C13—H13A	109.5
C2—C1—N1	118.25 (18)	C11—O2—C10	118.74 (14)
C2—C1—C6	122.08 (18)	C12—C10—C13	114.77 (19)
C2—C3—Cl1	119.27 (14)	H12B—C12—H12C	109.5
C2—C3—C4	121.10 (17)	H12B—C12—H12A	109.5
C3—C2—H2A	120.7	H12C—C12—H12A	109.5
C4—N2—H2	115.8	H13B—C13—H13C	109.5
C4—C3—Cl1	119.63 (14)	H13B—C13—H13A	109.5
C4—C5—H5	119.6	H13C—C13—H13A	109.5

### Hydrogen-bond geometry (Å, °)

**Table:**

*D*—H···*A*	*D*—H	H···*A*	*D*···*A*	*D*—H···*A*
N2—H2···Cl1	0.86	2.46	2.9328 (15)	115
N2—H2···O3	0.86	1.99	2.670 (2)	136
